# Pluronic F-68 and F-127 Based Nanomedicines for Advancing Combination Cancer Therapy

**DOI:** 10.3390/pharmaceutics15082102

**Published:** 2023-08-09

**Authors:** Nisar Ul Khaliq, Juyeon Lee, Sangwoo Kim, Daekyung Sung, Hyungjun Kim

**Affiliations:** 1 Department of Chemistry and Bioscience, Kumoh National Institute of Technology, 61 Daehak-ro, Gumi 39177, Republic of Korea; 2 Center for Bio-Healthcare Materials, Bio-Convergence Materials R&D Division, Korea Institute of Ceramic Engineering and Technology, 202 Osongsaengmyeong 1-ro, Osong-eup, Heungdeok-gu, Cheongju 28160, Republic of Korea; 3 Department of Chemical and Biomolecular Engineering, Yonsei University, 50 Yonsei-ro, Seodaemun-gu, Seoul 03722, Republic of Korea

**Keywords:** Pluronics, micelle, gelation, cancer nanomedicines, combination therapy

## Abstract

Pluronics are amphiphilic triblock copolymers composed of two hydrophilic poly (ethylene oxide) (PEO) chains linked via a central hydrophobic polypropylene oxide (PPO). Owing to their low molecular weight polymer and greater number of PEO segments, Pluronics induce micelle formation and gelation at critical micelle concentrations and temperatures. Pluronics F-68 and F-127 are the only United States (U.S.) FDA-approved classes of Pluronics and have been extensively used as materials for living bodies. Owing to the fascinating characteristics of Pluronics, many studies have suggested their role in biomedical applications, such as drug delivery systems, tissue regeneration scaffolders, and biosurfactants. As a result, various studies have been performed using Pluronics as a tool in nanomedicine and targeted delivery systems. This review sought to describe the delivery of therapeutic cargos using Pluronic F-68 and F-127-based cancer nanomedicines and their composites for combination therapy.

## 1. Introduction

**Figure 1:**
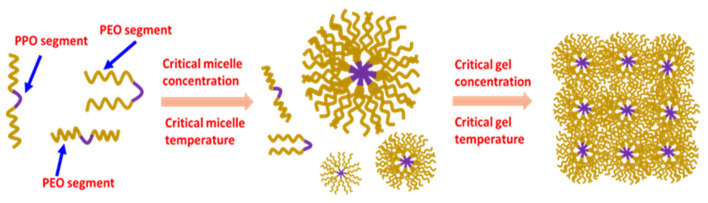
Micelle and gel formation exhibited by Pluronics in the aqueous medium.

Pluronics^®^ are Poloxamers composed of triblocks of poly (ethylene oxide) (PEO)–poly (propylene oxide) (PPO)–poly (ethylene oxide) (PEO). They are the most commonly and frequently used steric stabilizers for formulations of nanostructured and/or nanosized drug delivery tools due to their commercial availability and low cost [1,2,3]. These poloxamers are amphiphilic due to the central hydrophobic PPO segment’s connection to two side chains of hydrophilic PEO. As a result, the copolymers self-assemble in aqueous media and transform into core-shell micelles. These micelles have a diameter ranging from 10 to 100 nm with a central hydrophobic core (PPO block) connected to hydrophilic shells (PEO blocks) [4,5,6,7]. Pluronics are versatile owing to their multiple combinations of molecular weights and commercially available PEO/PPO ratios. Due to their flexible physiochemical characteristics, a Pluronic micelle can be further modified based on its therapeutic cargo and is considered an important class of biomedical polymer [8]. The micellar structure of a Pluronic micelle is displayed in Figure 1. The formation of the micellar structure and subsequent gelation by Pluronics are concentration- and/or temperature-dependent phenomena. When the concentration increases above the critical micelle concentration and critical micelle temperature, Pluronic micelles are formed. A significant reduction in the critical micelle concentration is reported to occur with even a small temperature change. This effect is due to the variance in the hydration of the PEO and/or PPO blocks with temperature change [9,10,11,12,13,14,15]. Increasing the concentration and/or temperature above the critical gel concentration (CGC) and critical gel temperature (CGT) changes the phase transition of the Pluronic solution to gelation. Therefore, the transformation of Pluronic micelles into gelation is due to the strong packing of the micellar structures, as shown in Figure 1.

Owing to the core-shell micellar nanostructures of Pluronics, the core of the nanomicelles is exploited to incorporate multiple diagnostics and therapeutics. However, the Pluronic shell inhibits the interaction of the incorporated cargo with the cells [16]. In addition to these features, Pluronics are pharmacologically active polymers that can modulate the response of cancer cells; therefore, they are primarily categorized as polymeric drugs. Pluronics have been reported to act as biological response modifiers at low critical micelle concentrations and are effective against multidrug-resistant (MDR) cancer cells. Pluronics are also effective at transporting drugs across cell barriers [6,7,17]. Based on other reports, the endocytosis of Pluronics changes the membrane microviscosity and inhibits drug efflux transporters, such as breast cancer resistance proteins (BCRPs), multidrug resistance proteins (MRPs), and P-glycoprotein (Pgp), at the cancer cell surface [18,19,20,21,22,23,24]. Pluronic micelles are composed of nonionic amphiphilic polymer surfactants that are biocompatible, non-toxic, and act as simple carrier systems. Pluronic micelles are stable in solution, even in the presence of salts or other destabilizing agents. Due to this stability, Pluronic micelles are simple and reliable drug delivery systems and are used to deliver multiple therapeutic cargos rather than a single regimen. Thanks to these properties, Pluronic micelles are considered versatile polymeric drug delivery systems.

## 2. Properties of Pluronic F-68 and/or F-127

**Figure 2:**
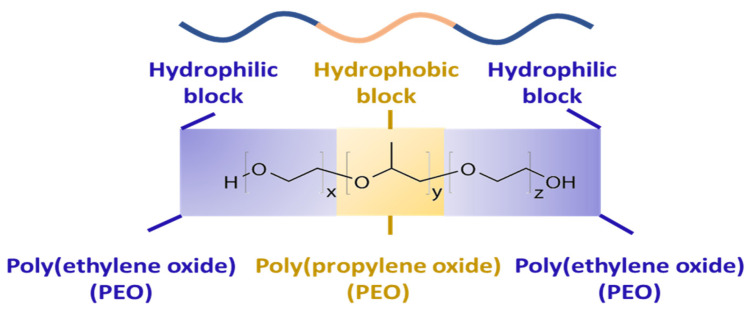
Chemical structure of a Pluronic. Y (in yellow) and X (in purple) are chains of propylene oxide (PO) and ethylene oxide (EO) blocks.

The different commercialized forms of poloxamer (Pluronic) are liquid (L), paste (P), and flakes (F) and are represented as L-92, P-105, and F-108. Pluronics are available in different physical states with different ratios of EO and PO units. The physical state of Pluronics is represented by letters such as L, P, and F. The first one or two digits (in three-digit numbers) multiplied by 300 represents the molecular weight of the hydrophobic (PO) segment and the last digit multiplied by 10 indicates the percentage of hydrophilic (EO) content. For instance, P-65 and P-105 are pastes and both have the number 5 at the end of their names. This indicates that both have a 50% EO (i.e., 5 × 10) content. Furthermore, the PO molecular weight of P-65 is 1800 g/mol (i.e., 6 × 300) and P-105 is 3000 g/mol (i.e., 10 × 300). The chemical structure of a Pluronic is described in Figure 2.

Different Pluronics vary in their physicochemical properties, such as molecular weight, PPO/PEO ratio, and hydrophobicity. For instance, Pluronic F-68, F-127, and P-123 are composed of PEO-PPO-PEO with block lengths of 80-30-80, 100-65-100, and 20-69-20, respectively. The hydrophilic corona of Pluronic F-68 and F-127 is four and five times longer than that of Pluronic P-123. This effect is due to the presence of higher EO block lengths. Different compositions and lengths of the blocks lead to varied micelle corona and both parameters affect the aggregation numbers and critical micelle concentration. The PPO/PEO ratio in Pluronics determines the hydrophilic–lipophilic balance (HLB) and clearly impacts the ability of Pluronics to self-aggregate. The PPO/PEO ratio is also relevant for the stability of the micelles and their compatibility and circulation time in the body. The use of Pluronics with high PEO proportions achieves highly stable micelles in water and better compatibility. The critical micelle concentration value of Pluronic F-127 was determined to be as low as 0.039 mg/mL, suggesting the high stability of the polymeric micelles upon dilution in the body. The lower critical micelle concentration value indicates a strong tendency toward the formation of aggregates and in turn, shows the high stability of micelles in solutions upon dilution. Furthermore, the critical micelle concentration values of P-85 and F-68 were measured and found to be 0.315 and 4.204 mg/mL, respectively. These findings show that the critical micelle concentration values were influenced by the length of the hydrophobic moiety. In other words, the longer the hydrophobic segment, the easier the micelle formation. The critical micelle concentration of Pluronic F-68 was found to be slightly higher than that of F-127 in an aqueous solution. Pluronic F-68 contains fewer hydrophobic PPO units in comparison to F-127. The HLB ratio of Pluronic F-127 and Pluronic F-68 are 22 and 29, respectively. The greater hydrophobicity of F-127 compared to F-68 can be accounted for by the smaller critical micelle concentration value of the former [25,26,27]. Likewise, Pluronic F-88 has 80% of its molecular weight (MW: 11,400) consists of the hydrophilic PEO, an HLB value of 28, a critical micelle concentration of 0.28% *w*/*w*, and a (PPO/PEO) ratio of 0.19. However, Pluronic F-127 (MW: 12,600) has an HLB value of 22, a critical micelle concentration of 0.004% *w*/*w*, and a (PPO/PEO) ratio of 0.33. The lower HLB value of F-127 and higher PPO/PEO are due to the increase in PPO blocks (65 units for F-127 vs. 39 units for F-88) which lead to the formation of stable micelles with more drug retention capacity than Pluronic F-88 [28].

Pluronic F-68 (poloxamer 188) and F-127 (poloxamer 407) are the only FDA-approved materials among Pluronics that are biocompatible and used in living organisms. They have been extensively studied and found to be safe for use in pharmaceutical and biomedical applications. However, other Pluronic polymers may not have FDA approval due to a variety of factors, including limited research data, insufficient evidence of safety or efficacy, or simply because they have not been extensively studied for a specific use. Different Pluronics vary in their physicochemical properties, such as molecular weight, PEO/PPO ratio, and hydrophobicity. These factors can influence the toxicity and biocompatibility of the polymer. As described above, variations in the block lengths among Pluronics and the higher PEO block lengths in Pluronic F-68 and F-127 provide a stealth effect [29,30]. As these Pluronics are biocompatible with tissues and exhibit a solution to gelation transformation, they are exploited for tissue regeneration. The gelation demonstrated by these forms of Pluronic is a three-dimensional scaffold that acts as a barrier between the host and transplanted cells [31,32,33,34]. Other forms of Pluronic variants such as P-105 contain a larger hydrophobic segment. This characteristic makes P-105 more suitable for solubilizing and stabilizing extremely hydrophobic substances, such as lipophilic or poorly water-soluble drugs. Therefore, it can be exploited as a solubilizer or a stabilizer agent in various nanomedicines. Likewise, another report suggests that Pluronic P-85 effectively inhibits the P-gp-mediated efflux of protease inhibitors [35].

**Figure 3:**
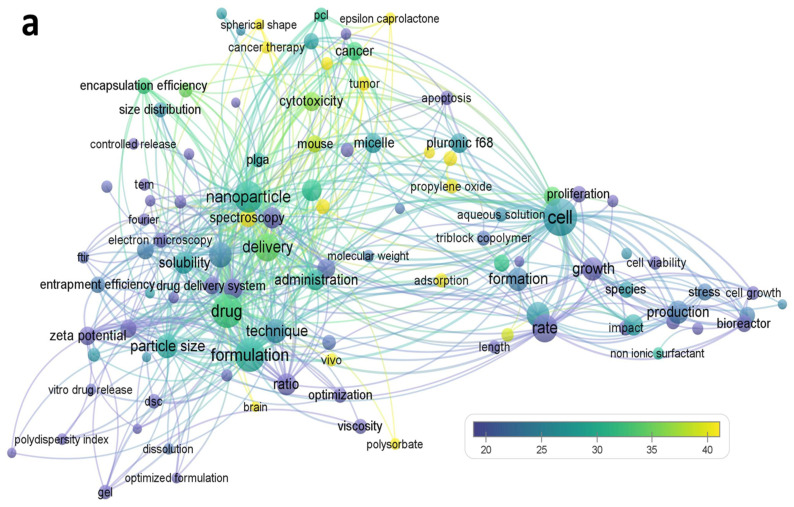

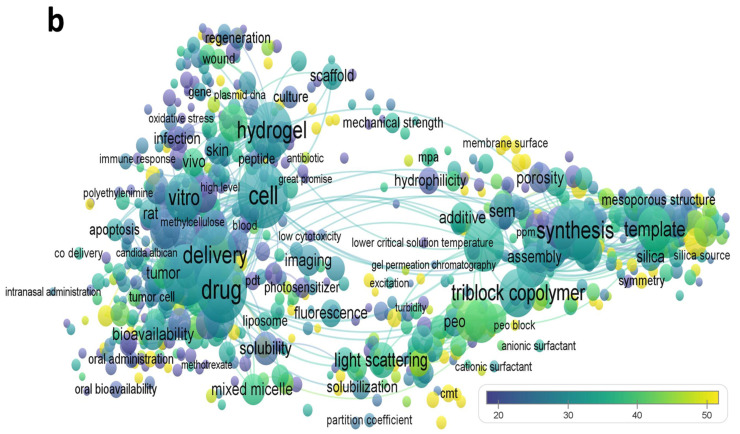
Bubble map showing the words mentioned in the publications on Pluronic F-68 (Publications: 841) and Pluronic F-127 (Publications: 3277) and associated drug delivery. The VOSviewer analytical tool was used to visualize recurring terms from titles and abstracts. A total of 107 and 698 terms were extracted from various publications associated with Pluronics (**a**) F-68 and (**b**) F-127 in the Web of Science. Colored circles represent the average citations of the terms.

Before describing the individual properties of Pluronics (F-68 and/or F-127), the keywords Pluronic F-68 and Pluronic F-127 were searched for in the literature. The list of Pluronic F-68 and Pluronic F-127-associated publications indexed in Web of Science was processed using the VOSviewer software to analyze and visualize recurring terminologies from titles and abstracts with a bubble map (see Figure 3). Word size represents the frequent use of terms that appear multiple times in a single manuscript and are counted as single words. The appearance of two words consecutively on the map indicates their multiple use in the articles. The colors on the bubble map represent the average citation of the terminologies.

Pluronic F-68 is a triblock copolymer composed of a central (PPO) segment connected to two (PEO) segments. The PPO and each PEO segment of Pluronic F-68 contain 25–30 units (average) and 75–85 EO units. The hydrophobic and hydrophilic segments impart a nonionic nature to Pluronic F-68 with surface-active characteristics and polymeric features [36]. Pluronic F-68 self-assembles in an aqueous solution at room temperature and transforms into a micellar structure. Pluronic F-68 is the only poloxamer that stabilizes injectable formulations of proteins and antibodies. Fourteen biological formulations using Pluronic F-68 have been generated. Among these, three are antibody formulations, and two are gene vector products [5,36].

Pluronic F-127 is a triblock amphiphilic copolymer and non-ionic surfactant comprising a central (PPO) block connected to two PEO blocks. The hydrophilicity of Pluronic F-127 is attributed to the presence of 70% PEO blocks. Pluronic F-127 is non-toxic, biocompatible, and stable, and enables in situ sol-gel transformations. Owing to these features, Pluronic F-127 is unique in drug delivery systems and applications [37,38]. In addition to these outstanding features, Pluronic F-127 exhibits some principal drawbacks such as rapid disintegration in bodily fluids and inadequate mechanical and bioadhesive characteristics [39]. Therefore, certain additives and biopolymers have been used to provide mechanical strength, viscosity, and bioadhesive capabilities to the Pluronic F-127 gelation system [40,41]. These biopolymers are cellulose derivatives; heparin, chitosan, and alginate are physically mixed with Pluronic F-127 to enhance its gelation properties. Similarly, salts play a pivotal role in providing mechanical strength to the gel system of Pluronic F-127. Moreover, release characteristics can be controlled by cross-linking certain chemicals with Pluronic F-127. For example, the controlled release behavior of ciprofloxacin was demonstrated using an admixture of Pluronic F-127 and hyaluronic acid, which exhibited a self-assembled network [39,42,43]. The USP specifications for Pluronic F-68 and F-127 are listed in Table 1.

The properties of the Pluronics in the preparation and pharmaceutical evaluation of Pluronic formulations are as follows.

### 2.1. Preparation of Formulation

For effective Pluronic dissolution and to limit possible alterations, the cold preparation method is preferred as compared to methods with high temperatures. A homogenous solution is prepared using Pluronic F-127 at 4–5 °C with other components (such as a drug) and cooled water. A 20–30% (*w*/*w*) Pluronic F-127 solution can be easily prepared, whereas a 35% (*w*/*w*) concentration solution needs to be frozen for a short period to liquefy the preparation [7,44,45]. To enable formulation stability, the pH and osmolarity are adjusted [46]. For the preparation of sterile formulations (ophthalmic or injectable), the sterilization of the Pluronic F-127 solution by autoclaving at 120 °C for 15 min is preferred [47,48]. As a result, the viscosity characteristics of the Pluronic F-127 solution are not greatly changed, which is interesting for the preparation of sterile formulations. For the Pluronic F-68 formulation with thymoquinone (TQ), the initial stock solution was prepared by dissolving it in dimethyl sulfoxide at 0.3, 0.7, and 1.1 mM concentrations and further dissolving in 1 mL acetonitrile. After stirring for 20 min, an aqueous solution of Pluronic-F68 was added and the mixture was stirred overnight. Following the evaporation of the organic solvent, the Pluronic F-68 formulation with TQ was prepared [49]. Likewise, Pluronic F-68 formulations with hydrophobic drugs such as docetaxel were also developed. After solubilizing docetaxel in Solutol and adding Pluronic F-68 at 60 °C for 10 min, a phase transition was induced. After cooling at 0 °C, the Pluronic F-68 formulation can be used for further experimental procedures [50].

### 2.2. Solubility Enhancement of Poorly Water-Soluble Agents

Pluronic F-127 improves the solubilization of poorly water-insoluble agents such as indomethacin and insulin [47,51,52,53]. Supplementing 22.5% *w*/*w* Pluronic F-127 in the aqueous medium enhanced the solubility of piroxicam 11-fold and appeared to be more effective than polysorbate or polyol. In the presence of 4% Pluronic F-127 in an aqueous medium (*w*/*v*), the solubility of nifedipine was 27-fold higher compared to water alone [54]. The intermolecular hydrogen bonding between nifedipine and Pluronic F-127 was revealed by infrared spectroscopy. It was also proposed that nifedipine is transformed to an amorphous state in crystalline Pluronic F-127 to improve its solubility. Additionally, a smoother nifedipine crystal surface was observed when a Pluronic F-127 solid dispersion was prepared by the melting method and physical mixing. Also, an increase in the dissolution rate of phenylbutazone was observed with solid dispersions containing Pluronic F-127 [44]. This property of Pluronic F-127 was further integrated with other engineering technologies to increase the dissolution of poorly water-soluble agents, such as spray freezing into liquid [55,56,57]. Danazol is also a poorly water-soluble agent and was incorporated in powders loaded with various polymers including Pluronic F-127. The spray-micronized porous powder contained amorphous danazol incorporated in a hydrophilic excipient. This formulation exhibited a complete dissolution in aqueous medium and molecular interactions between danazol and Pluronic were observed using scanning electron microscopy [58,59]. Likewise, Pluronic F-68 was used to increase the solubility of poorly water-soluble agents such as Emodin. In addition to its anti-inflammatory and wound-healing properties, Emodin also exhibited anti-tumor activity. The higher solubility of the Emodin was revealed with the increase in the concentration of Pluronic F-68. Pluronic F-127 also exhibited Emodin solubility; these forms of Pluronics were compared for their solubilizing properties and it was found that they could be used to increase the solubility of poorly water-soluble agents. Pluronic F-127 was more effective at concentrations greater than 10% due to its higher hydrophobicity and lower critical micelle concentration as compared to Pluronic F-68. When the Pluronic F-127 concentration was 20%, the solubility of emodin was 0.42 mg/mL. However, Pluronic F-68 at a 40% concentration exhibited 0.5 mg/mL of emodin solubility. Based on these findings, 20% F-127 or 40% F-68 was required to solubilize 0.5 mg/mL emodin [60,61].

### 2.3. Stabilization of Formulations

Pluronic F-127 stabilizes drugs including proteins. It decreases the tendency of peptide unfolding due to a low critical micelle concentration and the absence of electrostatic interactions. Pluronic F-127 maintains the structural integrity of proteins [62,63,64], and supplementing more Pluronic F-127 was seen to form micro-or nanoparticles. The hydrophilicity of protein-loaded poly(epsilon-caprolactone) microparticles was further increased with Pluronic F-127 and it was shown to help prevent the aggregation of these microparticles [65]. Similarly, Pluronic F-127 provides stability to urease-loaded poly (lactide-co-glycolide) microspheres and maintains their bioactivity. Also, certain interactions between Pluronic F-127 and liposomes exist and have been reported. For the preparation of liposomes, Pluronic F-127 was either co-solubilized with lipids or supplemented afterward in the already-formed liposomes. It also provided stability to liposomes in the liposomal delivery system and extended their half-life [66]. Interactions between the hydrophobic polypropylene block of Pluronics and the lipid bilayer exist and these interactions were confirmed by differential scanning calorimetry and photon correlation spectroscopy [67,68]. The size of vesicles was significantly reduced by Pluronic F-127 and confirmed by quasi-elastic light scattering. So, liposome stabilization is related to the incorporation or adsorption of Pluronic F-127, a phenomenon that occurs above the critical micelle temperature. However, below the critical micelle temperature, Pluronic F-127 molecules remain individual (non-associated or unimers). Pluronic F-68 also stabilizes parenteral protein/antibody formulations. The adsorption of protein to different interphases and formulations has been decreased with Pluronic F-68 as a surface-active agent for improving stability [36]. However, Pluronic F-68 also stabilizes proteins (lysozyme and recombinant granulocyte colony-stimulating factor (GCSF)) by surfactant–protein interactions. Grapentin et al. recently studied the impact of Pluronic F-68 on liquid mAb formulations and observed slightly visible particles in the formulations which were considered as mAb-dependent. Slightly increased Pluronic F-68 concentrations (0.05% vs. 0.02% (*w*/*v*)) were unable to prevent the occurrence of visible particles after storage for six to twelve months at 5 °C [69].

### 2.4. Strength of Gel

The in vitro determination of gel strength is helpful in developing a gel formulation with appropriate consistency and strength. For the measurement of in vitro gel strength, the gel is placed in a cylinder and submitted to a mass force using a piston. The time to sink down a predetermined distance through the formulation is representative of the gel strength [70,71]. The temperature (i.e., thermally reversible properties) and Pluronic F-127 concentration improve the gel strength and could be altered in the presence of drugs or additives. Pluronic F-127 gel is weakened by diclofenac, ethanol, and propylene glycol but strengthened by sodium chloride, sodium monohydrogen phosphate, and glycerine [70,71,72].

### 2.5. Adhesive Property

Bioadhesive properties are of great importance when prolonged residence time is required, particularly with topical formulations (such as rectal, cutaneous, or ophthalmic formulations). The bioadhesive force typically rises with gel strength, and its value is changed by the same factors (i.e., temperature and Pluronic F-127 concentration). Several solvents or ionic agents may change the adhesion properties of Pluronic formulations. Therefore, salts such as NaCl have been introduced to some Pluronic F-127 gels to increase residence time at the site of administration [72].

Determining bioadhesive properties is of great interest because leakage occurs often with rectal forms. Therefore, retaining the drug maximally in the rectum is an important consideration to prevent first-pass hepatic elimination. The visualization of the adhesive properties of Pluronic F-127 gel was performed by incorporating a colored marker (such as Ponceau S) into Pluronic F-127 gel [73]. Suppositories with a colorant (such as Blue n°1 lake 0.1% *w*/*w*) were administered in the rectums of rats to verify bioadhesive behavior and migration distance. This appeared to be an interesting predictive indicator of bioadhesive properties [71]. In general, the migration distance decreased with increasing mucoadhesive force; as a result, an area under the curve in plasma increased.

### 2.6. Solution to Gel Transformation Temperature

A decrease in the Pluronic F-127 concentration results in an increase in the sol-gel transition temperature. The sol-gel transition temperature (Tsol→gel) was measured using methods such as a glass microcapillary tube or a stirring magnetic bar. With this first method, after decreasing the temperature, the sample liquified and fell into the lower part of a glass tube. The (Tgel→sol) is equivalent to the (Tsol→gel) due to the reversible properties of thermogelation [74,75]. The second method involved gradually heating and stirring a solution of Pluronic F-127 constantly. Once the gel was formed, the magnetic bar stopped moving and the temperature displayed was considered as the Tsol→gel [71,76].

The bioadhesive forces are significantly modified by drugs or additives in such a way that when the Tsol→gel increases, the bioadhesive forces decrease and vice versa. These agents interrupt the Pluronic F-127 micellization and change the dehydration of hydrophobic PO blocks [77,78]. Agents such as diclofenac, ethanol, propylene glycol, and HCl decrease the gel strength and bioadhesive force and increase the Tsol→gel, whereas NaCl, Na_2_HPO_4_, and NaH_2_PO_4_ have the opposite effect [70,72]. As reported, adding sodium alginate, polycarbophil, or carbopol reduces the gelation temperature [71]. However, complex formulations such as cyclodextrins in Pluronic F-127 gel led to an increase in the Tsol→gel. This increase in the Tsol→gel is from disturbing the micellar packing and entanglements of Pluronic F-127 [76]. To increase the bioadhesive property and to modify the gelation temperature, the conjugation of 3, 4 dihydroxyphenyl-L-alanine (DOPA) moieties to Pluronic-F127 has been suggested. The Tsol→gel of Pluronic-F127 without modification generally ranges from 15 to 35 °C. On the contrary, the Tsol→gel of DOPA-Poloxamer 407 ranged from 22 to 31 °C, depending on the concentration. Since transparent gel was formed under physiological conditions by Pluronic F-127 solutions (20 wt.%), Pluronic F-68 as well as its mixtures with Pluronic F-127 formed gels only above the physiological temperature. The Tsol→gel of Pluronics F-68 and F-127 in aqueous media is 58 °C and 17 °C, respectively, when formed 25 wt% aqueous solutions [79]. Another report suggested that the Tsol→gel of a mixture of Pluronic F-68 and Pluronic F-127 in a 10:90 ratio is 29.3 °C [80]. Moreover, the Tsol→gel of a mixture of nanoparticles with Pluronic F-127 (80%) and Pluronic F-68 (20%) was approximately 33 °C [81].

## 3. Pluronic F-68 and Pluronic F-127 for Targeted Delivery of a Single Chemotherapeutic

Traditional chemotherapies are associated with major adverse effects owing to the lack of precise tumor targetability, non-specific toxicity, and drug resistance [82,83,84,85]. The challenges of conventional chemotherapies have been overcome using nanomedicine as a function of time [86,87,88]. For instance, Pluronic-based nanoparticles have been identified as potential nanomedicines for targeted chemotherapy. Pluronic F-68 and F-127 form self-assembled nanomicelles (10–100 nm) with a core-shell morphology in an aqueous medium. Therefore, Pluronic nanomicelle cores have been exploited as a storage area for diagnostics and therapeutics in targeted chemotherapy [5,16].

### 3.1. Pluronic-Based Micelles of Poorly Water-Soluble Anticancer Agents

Pluronic F-68-incorporated paclitaxel nanospheres with a core-shell morphology were developed using the “temperature-induced phase transition” method. The presence of PEO in Pluronic F-68 and its role in the surface coating of incorporated paclitaxel led to the development of nanospheres with extended plasma circulation and enhanced permeation and retention effect [89].

Generally, the route of administration determines the rate and extent of the absorption of Pluronic-based micelles into systemic circulation. After administration, Pluronic micelles enter the systemic circulation and are distributed throughout the body. Due to the small size of Pluronic micelles (10–100 nm), they can evade the reticuloendothelial system (RES) and avoid rapid clearance. The plasma circulation of Pluronic micelles can be prolonged by modifying their surfaces with hydrophilic polymers, such as polyethylene glycol (PEGylation). Pluronic micelles can also be distributed to various tissues and organs based on the size, surface properties, and physicochemical properties of the incorporated drugs. Pluronic micelles can accumulate in tumors through the enhanced permeability and retention (EPR) effect, ultimately releasing the encapsulated drug in a sustained manner. The FDA-approved drug Taxotere^®^ (commercial formulation of docetaxel with Tween 80) is clinically used; however, in addition to its serious side effects, its incompatibility with polyvinyl chloride has been highlighted. Fang et al. prepared Pluronic^®^ P-105 and F-127 mixed micelles with docetaxel and observed significant cytotoxicity in A549-Taxol-resistant cancer cells compared with cells subjected to Taxotere^®^ injections. Furthermore, these mixed micelles exhibited 1.85 times extended plasma circulation time and a 3.82 times larger area under the plasma concentration-time curve than Taxotere [90]. Following intravenous administration to Sprague–Dawley rats, the peak plasma concentration (Cmax) of Pluronic F-68 micelles containing paclitaxel was 188.0441 ± 51.3152 ng/mL compared to that of free paclitaxel (121.6545 ± 11.6474 ng/mL). Glycol chitosan and heparin were further introduced to modify the surface of Pluronic F-68 micelles containing paclitaxel (the composite micelles), which led to a Cmax of 339.1482 ± 16.2986 ng/mL. The plasma concentration-time curves from AUC0 to AUClast of free paclitaxel, Pluronic F-68 micelles containing paclitaxel, and the composite micelles revealed values of 85.0047 ± 7.5987 ng·h/mL, 179.782 ± 13.5721 ng·h/mL, and 394.5316 ± 74.5345 ng·h/mL, respectively [91]. The Pluronic F-68-based micelle groups maximally changed the pharmacokinetic parameters of paclitaxel compared to that of free paclitaxel. The Pluronic F-68-based composite micelles exhibited the largest Cmax and AUClast values. This effect was due to the prolonged plasma circulation of the composite micelles, which increased the targeting efficiency. The stealth effect plays a central role in nanomaterials for drug delivery applications by improving pharmacokinetics such as blood circulation, biodistribution, and tissue targeting [92]. One of the most important advantages of these Pluronic micelles is their hydrophilic PEO corona. Pluronics are PEG-based copolymers and, due to the presence of two PEO (PEG) blocks in their structure, prevent aggregation and protein adsorption, supporting the utility of steric stabilization in retarding RES clearance and leading thus to an increased blood circulation time (stealth effect) [93].

Docetaxel-loaded Pluronic F-68 nanospheres were developed to penetrate deeply into tumor tissues. Solutol was used as a solubilizer of docetaxel and Pluronic F-68 as an encapsulation material. The nanospheres exhibited a spherical morphology of 12 nm. During the drug loading phase, Nile Red was loaded into the docetaxel-loaded Pluronic F-68 nanospheres for tracking docetaxel in the tumor tissues. The disintegration of nanospheres in the acidic pH could be the driving force to release the drug. After endocytosis, the red signals in the cytoplasmic region of the tumor cells exhibited the presence of docetaxel. Because docetaxel targets tubulin, it stabilizes microtubules and thereby induces cell-cycle arrest and apoptosis [50].

### 3.2. Pluronic-Based Micelles of Water-Soluble Anticancer Agents

Doxorubicin (DOX) is a promising anticancer drug, however, its long-term usage induced cardiac damage and multidrug resistance (MDR) [94,95,96]. Pluronic F-68 and Pluronic F-127 triblock copolymers were employed to resolve this issue. Pluronic F-68-based micelles significantly shifted their biodistribution, leading to the enhanced accumulation of the micellar drug in tumor tissues compared to free DOX. Significant antitumor effects were observed in both DOX-sensitive and DOX-resistant cancer cells. After the intravenous injection of Pluronic F-68 into BALB/c nu/nu mice, the pharmacokinetic parameters were significantly improved, compared with that induced by free DOX. The AUClast values of free DOX, FRRG-DOX (a modified form of DOX, a pro-drug), and Pluronic F-68-FDOX (FRRG-DOX surface coated with Pluronic F-68) were 22.18 ± 4.52 µg·h/mL, 345.99 ± 68.9 µg·h/mL, and 1281.17 ± 240 µg·h/mL, respectively. Similarly, the plasma half-life (t_1/2_) values of free DOX, FRRG-DOX, and Pluronic F-68-FDOX were 1.33 ± 0.23 h, 7.96 ± 4.59 h, and 25.83 ± 0.80 h, respectively. The longest plasma t_1/2_ of Pluronic F-68-FDOX was due to the surface modification of Pluronic F-68 [97].

Pluronics^®^ L-61/F-127 mixed micelles containing DOX were employed in clinical trials of esophagus adenocarcinoma. These Pluronic micelles exhibited significant antitumor activity compared with the standard formulation of DOX [98,99]. Notably, this Pluronic micelle was the first Pluronic-based micelle formulation used for clinical investigation. This formulation was prepared by mixing DOX with Pluronic^®^ L-61 and F-127 micelles (SP1049C by Supratek Pharma Inc., Singapore). A phase II clinical trial of SP1049C for advanced gastroesophageal junction adenocarcinoma revealed a significant anticancer effect and highlighted its safety for such cancers [99,100]. Supratek Pharma Inc. carried out a clinical Phase 3 trial of SP1049C for the treatment of DOX-resistant cancer. In the United States, SP1049C was identified as an orphan drug for gastroesophageal carcinoma and was reported to participate in an international clinical Phase 3 study. Similarly, a clinical Phase 3 study of Mediclore^®^ (a Pluronic F-68/F-120-based thermo-sensitive Sol-Gel agent) for breast cancer surgery was also carried out; the axillary dissection is registered on https://clinicaltrials.gov (identifiers: NCT02967146; 5 January 2017). Surprisingly, clinical studies on Pluronic-based nanomedicines in oncology are scarce. Although many studies on Pluronic-based nanomedicines have been described in this review, their results must be clinically evaluated to provide new hope to patients with cancer.

Lee et al. developed a modified version of DOX called DEVD-S-DOX to counteract DOX-induced side effects. DEVD-S-DOX (a pro-drug) comprises DOX connected to DEVD (a tetrapeptide structure (aspartic acid-glutamic acid-valine-aspartic acid)) and is exploited to perform antitumor activity through radiation-induced apoptosis-targeted chemotherapy (RIATC). A Pluronic F-68 surface-coated DOX/DEVD-S-DOX nanocomposite was established for DOX-induced apoptosis-targeted chemotherapy (DIATC). The Pluronic F-68 nanocomposite-incorporated DOX/DEVD-S-DOX exhibited good targetability due to the enhanced permeability and retention effect and significant antitumor activity with substantially lower cardiotoxicity [101,102]. After endocytosis of the nanocomposite by the tumor cells and its subsequent disintegration due to acidic pH, free DOX initiates apoptosis with the expression of caspase-3, which in turn cleaves DEVD-S-DOX into free DOX. The DEVD-S-DOX released from the nanocomposite existed as a pro-drug form and the DOX entrance into the nucleus was restricted due to the lack of apoptosis-induced caspase-3 in the cells. Once caspase-3 is activated, DEVD-S-DOX is converted into active DOX to perform cytotoxic effects in the neighboring tissues.

Pluronic F-127 nanomicelles modified with biological ligands have been used for receptor-mediated targeting and the endocytosis of anticancer agents. DOX-incorporated Pluronic F-127/folic acid (FA)/D-α-tocopheryl polyethylene glycol succinate (TPGS) micelles were prepared via thin-film hydration to actively target ovarian (SKOV3) cancer cells. The nanomicelles exhibited spherical morphology with a hydrodynamic size of 173 ± 31 nm. The DOX-loaded nanomicelles exhibited enhanced cytotoxicity toward SKOV3 cells compared to the free form of DOX [103]. This effect was due to reduced drug efflux activity, enhanced cellular uptake, and the increased DNA binding of DOX after the DOX was released from the micelles in a pH-dependent manner. Similarly, transferrin (Tf)-conjugated DOX/Pluronic F-127/P-123 nanomicelles were developed to circumvent multidrug resistance in anticancer therapy. The nanomicelles had a size of 90.8 ± 2.1 nm with a spherical morphology. These nanomicelles hindered cell migration and changed the cell cycle sequences of various cancer cells. Furthermore, the nanomicelles exhibited significant tumor targetability and DOX delivery in MDA-MB-231 tumor-grafted mice [104]. MDA-MB-231 are DOX-resistant breast cancer cells. A multimodal composite nanomicelle incorporating DOX with a surface-modified AS1411 aptamer was developed for active targeting therapy in human breast cancer. A nanomicelle system comprising aptamer-modified Pluronic F-127 and beta-cyclodextrin-linked poly(ethylene glycol)-b-polylactide was also mixed to improve DOX encapsulation and enhance nanomicelle stability. The nanomicelles exhibited a spherical morphology with a size distribution of 38.23 nm and nucleolin-mediated cell internalization at the cellular level. After intravenous injection, the nanomicelles displayed extended plasma circulation with significant antitumor efficacy and reduced cardiotoxicity in MCF-7 tumor-grafted mice [105].

Pluronic F-127-modified nanomicelles incorporating bufalin (an anti-tumor agent) were developed with thermal and redox-responsive properties. The bufalin-encapsulated cross-linked nanomicelles had a size distribution of 21 ± 3 nm with a spherical morphology. These nanomicelles displayed good uptake behavior and showed promise for preventing the proliferation of murine hepatocellular carcinoma (H22) tumor cells at the cellular level. After exhibiting their antitumor efficacy, the nanomicelles caused significant tumor suppression in H22 tumor-grafted mice with 100% survival until day 36 [106]. To deliver niclosamide to cisplatin-resistant lung cancer (A549) cells, Pluronic^®^ P-123/F-127 mixed micelles were developed. Pluronic^®^ F-127 and Pluronic^®^ P-123 were conjugated with biotin and rhodamine B for active targeting and bioimaging, and niclosamide was incorporated into the micelle core. Biotin-functionalized micelles were significantly endocytosed by A549 cells and exhibited significant toxicity to cisplatin-resistant lung cancer cells compared with non-biotin micelles. This effect was due to biotin receptor-mediated endocytosis which is highly expressed in A549 cancer cells [107]. Curcumin-incorporated Pluronic F-127 nanobodies have also been developed to target the overexpressed FLT3 receptors in leukemic cells. In vitro studies revealed that the nanobodies exhibited significant cytotoxicity in FLT3 overexpressing leukemic cells and were considered anti-leukemic targeted delivery vehicles [108]. A summary of the studies conducted in the last five years on Pluronic- based nanomedicines for cancer therapy is shown in Table 2.

## 4. Pluronic F-68 and F-127 Nanomicelles as Anticancer Combination Therapy

Cancer combination therapy is more effective than single therapy owing to the synergy between pharmacodynamics and less-needed therapeutic doses. Nonetheless, many patients with cancer do not achieve the desired results due to unsynchronized pharmacokinetics. Therefore, Pluronic-based delivery systems can be employed to regulate the premature release of therapeutic cargo and improve the pharmacokinetic profile. These delivery systems also serve as significant cancer combination therapies. Kelishady et al. prepared Pluronic F-127-based nanomicelles containing paclitaxel and co-delivered lapatinib for breast cancer metastasis [118]. Paclitaxel was exploited as a microtubule stabilizer, whereas lapatinib inhibited epidermal growth factor (EGFR). Pluronic with lapatinib blocked the efflux pumps. Moreover, a synergistic cytotoxic effect was achieved with paclitaxel. The nanomicelles had a size of 64.81 nm with a core-shell morphology based on transmission electron microscopy (TEM). Compared to free agents, nanomicelles exhibited significant in vitro cytotoxic effects in the T-47D cell line. The findings of this study provide a mechanistic approach to the combined effects of Pluronic micelles against various resistant cancer cells. To treat multidrug-resistant cancer, combination therapy using the chemotherapeutics DOX and paclitaxel has been exploited [119]. In fact, dual drug-loaded nanomicelles using the Pluronic-drug conjugates, P-105–DOX and F-127-paclitaxel, were developed. First, the P-105–DOX conjugate was developed and mixed with F-127-paclitaxel to form dual drug-loaded mixed micelles. This strategy entrapped paclitaxel in the core of dual drug-loaded mixed micelles. After the intravenous administration of mixed micelles into MCF-7/ADR tumor-grafted mice, enhanced antitumor activity with decreased cardiotoxicity was observed compared to that achieved with the free form of anticancer agents. Therefore, this strategy can be used in combination with various anticancer agents against resistant cancer cells.

**Figure 4:**
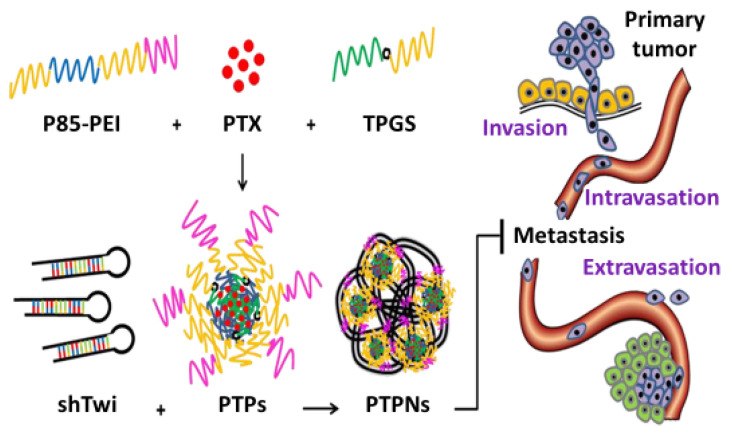
Schematic view of tumor growth suppression and metastasis prevention by PTPNs. Reprinted from [120]. Copyright © 2012 with permission from Elsevier.

In addition to Pluronic-based systems containing small molecules (anticancer agents) for combination therapy, chemical agents with RNA molecules (small interfering RNAs (siRNAs), microRNAs (miRNAs), and short-hairpin RNAs (shRNAs)) have been co-delivered using Pluronics for effective anticancer therapy. Shen et al. proposed a combination therapy comprising Pluronic nanomicelles for the co-delivery of paclitaxel and shRNA to simultaneously inhibit tumor proliferation and metastasis [120]. Pluronic P-85 (P-85), polyethyleneimine (PEI), D-α-tocopheryl polyethylene glycol 1000 succinate (TPGS), and paclitaxel were exploited to develop P85-PEI/TPGS/PTX (PTPs) nanomicelles. The PTP nanomicelles were further modified with shRNA to develop PTPNs. A schematic representation of tumor suppression and metastasis prevention is displayed in Figure 4. After intravenous injection, the PTPNs exhibited significant antitumor activity in 4T1 tumor allografts, suggesting enhanced nanomicelle accumulation in the tumor parenchyma via enhanced permeability and a retention effect. Pulmonary metastasis was analyzed in mice with 4T1 pulmonary metastasis treated with PTPN nanomicelles. After a histopathological study of the lungs, no metastasis nodules were observed in the lung tissues, thereby mimicking normal lung tissues. These findings revealed the integrity of the PTPN nanomicelles and their potential to treat metastatic breast cancer. Similarly, a treatment approach for hepatocellular carcinoma (HCC) was ascertained using siRNA and paclitaxel in combination therapy [121]. Pluronic F-68 was used as the polymeric material to develop nanobubbles with paclitaxel and siRNA (PTX–NBs/siRNA). After tail vein injection, the ultrasound-sensitive (US) nanobubbles restricted tumor growth for 28 days. This restriction was due to the penetration capability and synergistic activity of paclitaxel and siRNA. The mechanism of such synergistic activity is due to the reverse of paclitaxel-associated drug resistance and simultaneous antiapoptosis-related drug resistance of siRNA-targeted (BCL-2 siRNA) delivery. Rafael et al. used Pluronic F-127 and gelatin to develop nanoscale cetuximab-conjugated micelles (PM) for the delivery of siRNA into breast cancer cells [122]. Breast cancer cells are enriched with epidermal growth factor receptors (EGFRs), which are potential targets for anticancer drugs. To enhance receptor-mediated endocytosis, the Pluronic F-127 surface of the micelles was functionalized with cetuximab. Gelatin was used to provide cationic binding moieties (ζ = +30 mV) to form a complex with the siRNA. The nanomicelles had a size of 40 nm and exhibited a spherical morphology. Micelle formation using this approach is an alternative protocol for the safe delivery of genes, siRNA biomolecules, or pharmaceuticals as combination therapy for breast cancer. Overall, Pluronic nanomedicines exhibited significant antitumor synergism.

## 5. Pluronic F-68 and Pluronic F-127 Nanomicelles for Chemo-Photodynamic Combination Therapy

**Figure 5:**
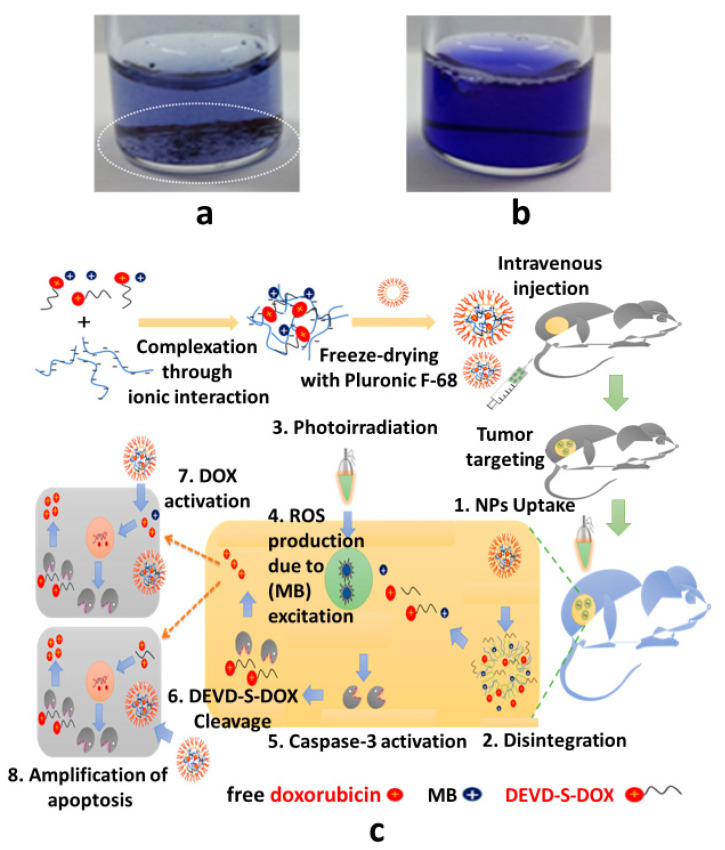
(**a**) MB and DEVD-S-DOX, (**b**) MB/DEVD-S-DOX/F-68 NPs in the aqueous medium, (**c**) chemo–photodynamic combination cancer therapy. Reprinted with permission from [123]. Copyright © 2018 American Chemical Society.

Combination therapy for cancer is accomplished using nanomedicine chemotherapy and photodynamic therapy. Khaliq et al. developed Pluronic F-68 nanomicelles for chemotherapy or photodynamic combination therapy via caspase-3 activation using photoirradiation [123]. Anionic heparin and cationic DEVD-S-DOX (pro-drug) or methylene blue (MB) were mixed to form a single assembly via ionic bonding. Additionally, the complex mixture was stabilized using Pluronic F-68 via freeze-drying (see Figure 5a,b). After intravenous injection, the nanomicelles accumulate in the tumor parenchyma through the enhanced permeability and retention effect. Upon laser irradiation, MB generates reactive oxygen species (ROS), which stimulate caspase-3, leading to the disintegration of DEVD-S-DOX into active DOX to exert cytotoxic effects in neighboring tissues. Thus, a combination of chemotherapy and photodynamic therapy (PDT) is successfully directed to the tumor area by MB or DEVD-S-DOX release using Pluronic F-68 nanomicelles following photoirradiation (Figure 5c).

To treat melanoma, a chemo-photodynamic combination therapy was developed using DOX-loaded pheophorbideA (PheoA)-modified Pluronic F-127 micelles [124]. To develop the micelles, a photosensitizer (PheoA) was initially introduced into the hydrophilic PEO segment of F-127-PheoA. Thereafter, DOX was incorporated into the hydrophobic core of F-127-PheoA to obtain double drug-loaded micelles for combination therapy. The nanomicelles were spherical structures with a size distribution of 146.5 nm. Upon photoirradiation at the cellular level, the PheoA in the nanomicelles generates ROS, and combination therapy is established with DOX release from the micelles. An intravenous injection of DOX/F-127-PheoA micelles following photoirradiation caused significant tumor shrinkage (73.5%) in B16 tumor-grafted mice compared to the nanomicelles following no irradiation. These findings highlight the importance of DOX/F-127-PheoA nanomicelles as an efficient combination therapy for melanoma.

**Figure 6:**
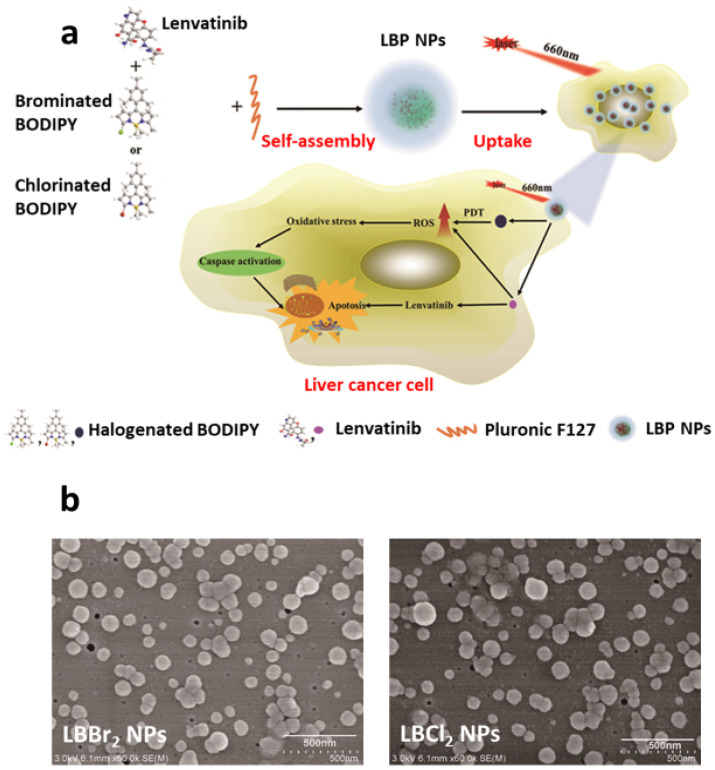
(**a**) Schematic illustration of self-assembled nanoparticles with Lenvatinib and Halogenated BODIPY. (**b**) Scanning electron microscope images of LBBr_2_ nanoparticles and LBCl_2_ nanoparticles. Reprinted from [66]. Copyright © 2021 Zong et al.

Zong et al. employed pH-responsive nanomedicines with chemophotodynamic capabilities to treat liver cancer [125]. Briefly, halogenated variants of boron-dipyrromethene (BODIPY) (a photosensitizer), such as brominated-BODIPY and chlorinated-BODIPY were combined with a chemotherapeutic agent (Lenvatinib) following a nanoprecipitation approach to develop a multifunctional nanomedicine. The self-assembled nanomedicine was then incorporated into Pluronic F-127 to develop LBP nanoparticles (Figure 6a), which exhibited significant stability in an aqueous medium. Two halogenated BODIPYs were used to prepare the nanoparticles, which were designated as LBBr_2_ and LBCl_2_ nanoparticles before encapsulation in Pluronic F-127. Both nanobodies exhibited a spherical morphology (see Figure 6b), with a size distribution of 75 ± 1.6 nm for the LBBr_2_ nanoparticles and 85 ± 2.4 nm for the LBCl_2_ nanoparticles. After encapsulation into Pluronic F-127, the nanoparticles became core-shell LBP nanoparticles. Pluronic F-127 acts as a shell, providing stability to the core of nanoparticles containing halogenated BODIPY with Lenvatinib during plasma circulation. Upon photoirradiation, the LBP nanoparticles generated ROS in the acidic environment of liver cancer cells, significantly improving the molecular targeting efficiency of Lenvatinib. Therefore, the photoirradiated LBP nanoparticles exhibited a remarkable inhibition of liver cancer cell growth. Both BODIPY and Lenvatinib have numerous clinical applications. Thus, the LBP nanomedicine can not only provide benefits to patients with hepatocellular carcinoma but can also be exploited for cancer diagnostics.

Pluronics (F-68 and F-127) have also been employed in photodynamic/photothermal and/or radiotherapy/chemotherapy combination therapy. Ma et al. prepared Pluronic F-127 nanocomposites with MB and graphene oxide (GO) for photodynamic/photothermal combination therapy [126]. First, aqueous solutions of GO and MB were mixed to develop a self-assembled GO-MB nanocomposite. Subsequently, the nanocomposite was incorporated into Pluronic F-127 to develop GO-MB/PF-127 nanocomposites using the film hydration method. The nanocomposites had high stability in aqueous media owing to the presence of Pluronic F-127 on the surface. After the endocytosis of the GO-MB/PF-127 nanocomposites by SiHa cells, anticancer combination treatment was administered using photodynamic/photothermal therapy. After photoirradiation of the nanocomposite, MB generated ROS and induced apoptosis; meanwhile, GO exhibited a photothermal effect to enable a combined effect. MB and GO are among the most potential therapeutic agents and have gained significant attention in clinical practice [127,128,129,130,131]. In fact, a significant cytotoxic effect was revealed in SiHa cells using anticancer combination treatment compared to PDT with PTT alone. Ma et al. determined the theranostic activity of DOX-incorporated complex core-shell nanomicelles [132]. Pluronic F-127, peptide-amphiphile pal-AAAAHHHD (PA), and DOX were mixed in deionized water, and triethylamine was added dropwise for complexation. Subsequently, GdCl3·6H_2_O was mixed with the complex micellar mixture to form DOX-incorporated hybrid nanomicelles. The core of the nanomicelles encapsulated DOX, and the shell of the nanomicelles comprised Gd (III)-chelates, PEO, and peptide. After intravenous injection of Pluronic F-127-based hybrid nanomicelles (DOX equivalent = 4.76 mg per kg and Gd = 20 µmol per kg), a combination effect based on theranostic functionality was ascertained in tumor-bearing mice. Similarly, Pluronic F-68 coated gold nanoparticles and DOX were incorporated into vesicles to produce vesicle nanoparticles [133]. After freeze-drying with Pluronic F-68, core-shell nanoparticles were formed and used for radio–chemotherapy combination therapy. TEM revealed a spherical nanostructure with a size distribution of 206 ± 10.11 nm. After the intravenous injection of the Pluronic F-68 core-shell nanoparticles, significant antitumor efficacy was observed in SCC-7 tumor-grafted mice treated with radiation (5 Gy) and 2 mg/kg DOX. This efficacy was due to the combined effects of radiotherapy and chemotherapy.

## 6. Pluronic F-68- and Pluronic F-127-Based Hydrogels as Cancer Combination Therapy

Hydrogels and nanogels can open new avenues for the use of nanotechnology in innovative cancer therapies. Hydrogels are 3D polymeric structures with the highest water absorption capacity and comprise materials that exhibit biocompatibility or biodegradability. Such materials can be acquired naturally or synthetically and integrated into three-dimensional networks via physical or chemical intermolecular interactions. Notably, these materials are extensively used in biomedical, biotechnological, and drug delivery systems for cancer treatment [134,135,136,137]. The use of Pluronic F-68 and F-127-based hydrogel systems as anticancer combination therapies is presented below.

**Figure 7:**
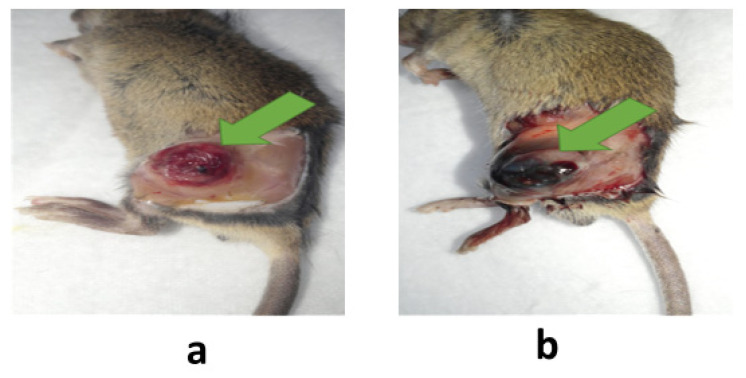
Intratumoral injection of (**a**) methylene blue and DEVD-S-DOX mixture and (**b**) F-68/methylene blue and F-127/DEVD-S-DOX nanomedicine mixture which transform into a gel at the tumor site. The arrows represent the comparison of gel formation at the tumor sites after intratumor injections. (**a**) No gel formed (**b**) Gel formed. Reprinted from [81]. Copyright © 2017 with permission from Elsevier.

Khaliq et al. developed temperature-sensitive Pluronic nanoparticles (with F-68 and F-127) as an anticancer combination therapy using a photoinduced apoptosis-targeted chemotherapy (PIATC) approach. The Pluronic nanomedicines, DEVD-S-DOX (using F-127) and MB (using F-68), were prepared via freeze-drying. A mixture of these nanomedicines forms a solution at room temperature and undergoes gelation at 37 °C [81,138]. Notably, the Pluronics (F-68 and F-127) were transformed into gel at 58 °C and 17 °C in the aqueous medium [79]. Nanoparticles incorporating MB or DEVD-S-DOX were surface-coated with Pluronics (F-68 and F-127). Therefore, the transformation capability of these nanomedicines into gel could be observed at 37 °C. After intratumoral injection, the Pluronic F-68 and F-127 nanomedicine mixture in the solution state was transformed into a gel (see Figure 7) to serve as a combination therapy in tumor tissues using the PIATC approach. After disintegration, the gel in the tumor tissues released MB and DEVD-S-DOX. Upon laser irradiation, MB induces the expression of caspase-3 by ROS, which transforms DEVD-S-DOX into active DOX which performs induced apoptotic chemotherapy in the neighboring tissues.

Hu et al. prepared an in situ thermosensitive sol-gel using paclitaxel and lapatinib, which exerted an anticancer combination effect [139]. First, paclitaxel nanoparticles and lapatinib microparticles were prepared and mixed with Pluronic F-127 (20% *w*/*w*). The mixture was transformed into a gel at 37 °C. After local injection around the MCF-7/ADR cell-grafted mice, the mixture became a gel due to physiological temperature. A mechanistic approach for the combined anticancer effect was designed and exploited to deliver paclitaxel (for a short period) and lapatinib (for a long period) to tumor tissues. The erosion of nanoparticles and microparticles from the gel structure and the co-delivery of chemotherapeutics into tumor tissues led to a combination of anticancer effects. The study revealed a safety profile, in addition to significant antitumor efficacy. Thus, this co-delivery could be considered an alternative protocol for combination therapy in clinical practice.

## 7. Conclusions and Future Perspectives

Pluronic F-68 and F-127 are versatile triblock copolymers that form micelles and gels. Pluronics with several anticancer agents and drug molecules have been exploited for cancer diagnosis and therapy. In this review, F-68- and F-127-based systems for the tumor-targeted delivery of a single chemotherapeutic agent using active and passive targeting strategies were presented. In particular, Pluronic F-68- and F-127-based nanomicelles and hydrogel systems for the co-delivery of chemotherapeutics, photodynamic, and photothermal agents, and radiotherapeutics and chemotherapeutics as cancer combination therapy were described. Notably, a mixture of these Pluronics and other biopolymers can be exploited as effective combination therapies. In addition, the F-68 and F-127 segments can be modified with targeting modalities to further increase their targeting efficiency as effective combination therapies for cancer and other diseases.

Polymeric micelles such as the polymer–cisplatin complex micelle (NC-6004) were formed by the poly(ethylene glycol)–poly(aspartic acid) block copolymers and cisplatin and utilized for a tumor-targeted drug delivery system. These polymeric micelles had a size of 28 nm and were found to be very stable in an aqueous medium in long-term storage. NC-6004 reached clinical development and showed some encouraging results. Likewise, the polymer–platinum conjugate (AP5280) is an N-(2-hydroxypropyl) methacrylamide (HPMA) copolymer-bound platinum (Pt). The DOX was coupled to the HPMA polymer through a tetrapeptide linker (glycine (Gly)–phenylalanine (Phe)–leucine (Leu)–glycine (Gly) or GFLG) which is susceptible to cleavage by lysosomal protease. This AP5280 exhibited superior stability in an aqueous medium and reached clinical development. A polymeric pro-drug conjugate poly[N-(2-hydroxypropyl) methacrylamide]–GlyPheLeuGly–DOX is the first clinically investigated enzyme-responsive system. The DOX became active by the cleavage of the peptidyl linker of GPLG through cathepsins in the lysosome [140].

In comparison to the above-mentioned polymer drug conjugates in clinical systems, a Pluronic micelle is a nonionic polymer with a high self-assembling property. This capability of Pluronics encourages the use of a lesser amount of Pluronic surfactants to form micelles, observing a low critical micelle concentration value and forming a core-shell micelle system, in addition to versatility while transferring from the bench to the clinic. Pluronic F-68 micelles previously faced certain issues associated with the high drug loading of poorly water-soluble drugs. For example, to increase the encapsulation of poorly water-soluble drugs such as docetaxel into the core of Pluronic F-68 nanoparticles, liquid PEG was used as a solubilizer for docetaxel and Pluronic F-68 was used as the encapsulation material. Because a temperature-induced phase transition at 100 °C for 30 min was used, oxidation of docetaxel occurred, and the diameter of the prepared nanoparticles became approximately 150 nm. Solutol was used as a solubilizer for docetaxel instead of PEG to perform the temperature-induced phase transition at 60 °C for 10 min to overcome these difficulties. The temperature-induced phase transition at 60 °C for 10 min produced stable docetaxel-loaded Pluronic nanoparticles with a diameter of 12 nm. As Pluronic F-68 mainly coated the surface of nanoparticles and PEO was the main component of the Pluronics, the prolonged retention of Pluronic nanoparticles was expected in systemic circulation, which is a prerequisite for the extended permeation and retention (EPR) effect. Further stability was provided to docetaxel-loaded Pluronic NPs by incorporating them into vesicles (liposomes) to form the vesicle NPs. Additional stability was provided to vesicle NPs by surface coating with Pluronic F-68 through lyophilization [50,141].

Pluronic-based nanomedicines have shown promising results in preclinical studies on drug delivery applications owing to their unique physicochemical properties, biocompatibility, and biodegradability. According to some studies, repeated exposure to Pluronic-based nanoparticles induces an immune response that leads to the production of antibodies against the polymer. These antibodies bind to and clear subsequent doses of the nanoparticles, thereby reducing their effectiveness. Also, the variability in the physical and chemical properties of Pluronic-based nanoparticles can affect their stability, drug release kinetics, and targeting efficiency. However, the shortcomings of Pluronic-based nanomedicines can be resolved for effective clinical translation. As Pluronic F-68 and F-127 are United States (U.S.) FDA-approved Pluronics, F-68- and F-127-based nanomedicines show great promise for drug delivery applications.

In the future, we will explore other strategies that can be utilized as combination therapies to achieve significant treatment outcomes. Pluronic F-68- and F-127-based nanomedicines can be used as stimuli-responsive drug delivery systems. Highly potent anticancer agents, such as DOX, and/or other anticancer agents can be modified into pro-drugs with minimal nonspecific toxicity. These pro-drugs are enzyme-activated peptide-drug conjugates that are converted into active drugs upon specific enzyme expression in response to specific stimuli and include light-triggered caspase-3 activatable pro-drugs used as anticancer agents. Notably, these pro-drugs have been incorporated into Pluronic F-68 or F-127 to synthesize core-shell nanomedicines. After endocytosis by tumor tissues, the pro-drugs are released from the nanomedicines and converted into active drugs upon caspase-3 expression via photoirradiation.

Future nanomedicines comprising F-68 or F-127 should be developed to incorporate and deliver complex molecules with significant anticancer activities. For example, some products approved by the U.S. FDA, such as Oncaspar^®^, Abraxane, and Kadcyla, have been previously compared with other formulations [142,143,144,145]. The currently marketed products are relatively limited but can be expanded in the future after new formulations enter clinical trials. Notably, Pluronic nanomedicines can be employed as chemoimmunotherapy or photodynamic immunotherapy. Therefore, F-68 or F-127 based nanoformulations can be developed to kill cancer cells and trigger the immune system to fight cancer. For example, photosensitizers and/or chemical agents in Pluronic F-68/F-127-based nanomicelles or nanogels have been used as combination therapy.

In addition to excellent targeting capabilities and functionalities across tumor tissues shown by various micelles-based nanomedicines, some shortcomings exist. To avoid repetitive injections of micelles, and provide the required concentration of drugs at once for sustained delivery, a topical or injectable gel for physical targeting should be considered as it has additional advantages over passive or other actively targeted therapies. The gel can deliver a drug throughout the tumor regardless of vascular status, thus providing accurate dosing without systemic toxicity. A report suggested that 85% of the reported stealth nanomaterials/drug delivery systems encounter a sharp α-phase clearance in blood circulation, that is, a rapid drop of blood concentration to half of the administered dose within 1 h post administration [92]. In fact, whether for tissue-targeting or cell-targeting, nanomaterials/drug delivery systems are difficult to navigate in a complex biological environment. Among the complexity, cross-vasculature transport, cross-tissue transport, and cross-cell membrane transport are the major barriers. These biological barriers complicate the prediction of pharmacodynamics by blood pharmacokinetics [92].

Immunotherapy has emerged as a promising strategy for cancer treatment, in which durable immune responses have been generated in patients with malignant tumors. Cancer immunotherapy includes five main classes, immune checkpoint blockade (ICB) therapy, lymphocyte-promoting cytokine therapy, chimeric antigen receptor T-cell (CAR-T) therapy, agonistic antibodies, and cancer vaccines [146]. Biomaterials have played vital roles as smart drug delivery systems for cancer immunotherapy to achieve both enhanced therapeutic benefits and reduced side effects. Recently, a cell-loading injectable hydrogel scaffold has been produced for tissue regeneration [147]. Fang et al. designed a PDT-motivated ATV (P-ATV) in a Fmoc-KCRGDK–phenylboronic acid (FK–PBA) hydrogel, which mobilizes local immune activation to inhibit the relapse of postoperative tumors [148]. Kim et al. developed a copolymer hydrogel system from gelatin and Pluronic^®^ F-127 that is widely used in humans to enable the sustained release of a nitric oxide donor and antibody-blocking immune checkpoint cytotoxic T lymphocyte-associated protein-4 for efficient and durable anti-tumor immunotherapy [149]. Pluronic F-127 is a clinical biomaterial and makes a thermosensitive hydrogel. Therefore, it is quite reasonable to make a cell-loading injectable hydrogel scaffold with Pluronic F-127 which can be used as a template for postsurgical tumors and personalized immunotherapy. PDT can not only kill tumor cells directly but also induce immunogenic cell death (ICD), which provides antitumor immunity [150]. After applying PDT, the Pluronic F-127 thermosensitive hydrogel mobilizes local immune activation to inhibit the relapse of postoperative tumors. This could be a promising approach for future development to achieve combination therapy via PDT-driven cancer immunotherapy as well as provide new perspectives on the treatment of cancer. 

## Figures and Tables

**Table 1:** Pluronic F-68 and F-127 specifications in the USP.

Pluronic	Physical State	Mol. Weight (Average)	EO Segments (Each Segment; USP)	PO Segment (USP)	% Weight EO (USP)	Reference
F-68	Solid	7680–9510	12	27	81.8 ± 1.9	[36]
F-127	Solid	9840–14,600	101	56	73.2 ± 1.7	[36]

Pluronics have a single PO segment and double EO segments.

**Table 2:** Pluronic-based nanomedicines for cancer therapy (single chemotherapeutic carrier).

Pluronic-Based Nanomedicines	Drug Incorporated	Cancer Cell Treatment	Benefits	References
Pluronic F-127 and phenylboronic ester-grafted (PHE)-Pluronic P-123-based micelles	Doxorubicin	(MCF-7/ADR) Breast cancer cells	Doxorubicin efflux and detoxification for efficiently reversing breast cancer resistance	[109]
Pluronic P-123 modified with α-tocopheryl succinate-based micelles	Doxorubicin	(MCF-7/ADR) Breast cancer cells	Improved the delivery of fluorescent dyes and protein across the blood–brain barrier (BBB)	[110]
Pluronic F-127- and P-123-based micelles	Doxorubicin	(MCF-7/ADR) Breast cancer cells	Efficiently overcame MDR in breast cancer	[111]
Pluronic P-123-PEG2000-DSPE-based micelles	Doxorubicin	(MCF-7/ADR) Breast cancer cells	Enhanced tumor-suppressing effect on drug-resistant breast cancer cells	[112]
Pluronic P-127 and vitamin E-TPGS-based micelles	Resveratrol	(MCF-7 and MDA-MB-231) Breast cancer cells	Effective at selectively targeting aggressive forms of breast cancer	[113]
Pluronic F-127 conjugated to ALN-based micelles	Curcumin	Osteolytic tumors	Effective and targeted delivery of curcumin to osteolytic tumors in bone	[114]
Pluronic F-68-GL44 galactosylated-based micelles	Harmine	Liver cancer cells	Significant improvement in oral bioavailability of Harmine and drug targeting capability	[115]
Pluronic F-127/N,N,N trimethyl chitosan-based hydrogel	Docetaxel	(U87MG) Brain tumor cells	Enhancement in the sustained release behavior of DTX and inhibited the orthotropic glioblastoma tumor	[116]
Pluronic P-123-antiGPC3/TPGS-b-PCL Aptamer	Sorafenib	Hepatocellular carcinoma	Significantly improved the targeted therapy of liver cancer.	[117]

## Data Availability

Not applicable.

## Abbreviations

PEO: polyethylene oxide; PPO: polypropylene oxide; CGC: critical gel concentration; MDR: multidrug-resistant; BCRPs: breast cancer resistance proteins; MRPs: multidrug resistance proteins; SCC7: squamous cell carcinoma-7; DOX: doxorubicin; MDR: multidrug resistance; CGT: critical gel temperature; DEVD: aspartic acid-glutamic acid-valine-aspartic acid; Tf: transferrin; FLT3: fms related receptor tyrosine kinase 3; TEM: transmission electron microscope; siRNAs: small interfering RNAs; miRNA: microRNA; shRNA: short-hairpin RNA; PEI: polyethyleneimine; EGFR: epidermal growth factors; TPGS: D-α- Tocopheryl polyethylene glycol 1000 succinate; PA: pal-AAAAHHHD; PIATC: photo-induced apoptosis-targeted chemotherapy; PTX: paclitaxel; BODIPY: Boron-dipyrromethene; GO: graphene oxide; HCC: hepatocellular carcinoma; US: ultrasound-sensitive; MB: methylene blue; ROS: reactive oxygen species; PheoA: pheophorbideA; EPR: extended permeation and retention; DIATC: doxorubicin induced apoptosis-targeted chemotherapy.
